# Supplementary material for: Endogenous retroviruses are a source of enhancers with oncogenic potential in acute myeloid leukaemia
Source: Nat Commun. 2020 Jul 14;11:3506. doi: 10.1038/s41467-020-17206-4 (PMC7360734; doi:10.1038/s41467-020-17206-4)
Supplement: Supplementary file 3 — Description of Additional Supplementary Information [file 41467_2020_17206_MOESM3_ESM.pdf]

Description of Additional Supplementary Files

File Name: Supplementary Data 1
Description: Results of DHS permutation test for each repeat family.

File Name: Supplementary Data 2
Description: List of AML-associated spliced transcripts emanated from A-DARs.

File Name: Supplementary Data 3
Description: K562-enriched TFs that are bound to A-DARs.

File Name: Supplementary Data 4
Description: TF motif frequency within A-DARs with and without DHSs.

File Name: Supplementary Data 5
Description: Results from the analysis of motif enrichment (MEME suite).

File Name: Supplementary Data 6
Description: Differentially expressed genes in upon CRISPRi.

File Name: Supplementary Data 7
Description: List of ERV elements that display regulatory activity in myeloid leukemia.

File Name: Supplementary Data 8
Description: Primers used in this study.

File Name: Supplementary Data 9
Description: External datasets used in this study.
