# Supplementary material for: Endogenous retroviruses are a source of enhancers with oncogenic potential in acute myeloid leukaemia
Source: Nat Commun. 2020 Jul 14;11:3506. doi: 10.1038/s41467-020-17206-4 (PMC7360734; doi:10.1038/s41467-020-17206-4)
Supplement: Supplementary file 13 — Reporting Summary [file 41467_2020_17206_MOESM13_ESM.pdf]

nature research

Corresponding author(s): Özgen Deniz, Miguel R. Branco

Last updated by author(s): May 29, 2020

Reporting Summary

Nature Research wishes to improve the reproducibility of the work that we publish. This form provides structure for consistency and transparency in reporting. For further information on Nature Research policies, see Authors & Referees and the Editorial Policy Checklist .

Statistics

For all statistical analyses, confirm that the following items are present in the figure legend, table legend, main text, or Methods section.

n/a	Confirmed	
☐	☒	The exact sample size (*n*) for each experimental group/condition, given as a discrete number and unit of measurement
☐	☒	A statement on whether measurements were taken from distinct samples or whether the same sample was measured repeatedly
☐	☒	The statistical test(s) used AND whether they are one- or two-sided<br>*Only common tests should be described solely by name; describe more complex techniques in the Methods section.*
☒	☐	A description of all covariates tested
☐	☒	A description of any assumptions or corrections, such as tests of normality and adjustment for multiple comparisons
☐	☒	A full description of the statistical parameters including central tendency (e.g. means) or other basic estimates (e.g. regression coefficient) AND variation (e.g. standard deviation) or associated estimates of uncertainty (e.g. confidence intervals)
☐	☒	For null hypothesis testing, the test statistic (e.g. *F*, *t*, *r*) with confidence intervals, effect sizes, degrees of freedom and *P* value noted<br>*Give P values as exact values whenever suitable.*
☒	☐	For Bayesian analysis, information on the choice of priors and Markov chain Monte Carlo settings
☒	☐	For hierarchical and complex designs, identification of the appropriate level for tests and full reporting of outcomes
☒	☐	Estimates of effect sizes (e.g. Cohen's *d*, Pearson's *r*), indicating how they were calculated

Our web collection on statistics for biologists contains articles on many of the points above.

Software and code

Policy information about availability of computer code

Data collection	High-throughput sequencing: NextSeq System Suite v2.1.2<br>qPCR: Roche LightCycler 480 Software v1.5<br>FACS: FACS Diva Software v6.1.3
Data analysis	Open source software: trim_galore! v0.4.5, Bowtie2 v2.1.0, MACS2 v2.1.1 , F-seq v1.84 , Hisat2 v2.0.5 , DESeq2, deepTools2.0, StringTie v1.3.3b, MEME SUITE v5.0.1, Seqmonk v1.42.0<br>Custom scripts used for data analysis are available at https://github.com/MBrancoLab/Deniz_2019_AML.

For manuscripts utilizing custom algorithms or software that are central to the research but not yet described in published literature, software must be made available to editors/reviewers. We strongly encourage code deposition in a community repository (e.g. GitHub). See the Nature Research guidelines for submitting code & software for further information.

Data

Policy information about availability of data

All manuscripts must include a data availability statement. This statement should provide the following information, where applicable:

- Accession codes, unique identifiers, or web links for publicly available datasets
- A list of figures that have associated raw data
- A description of any restrictions on data availability

High-throughput sequencing data that support the findings of this study have been deposited in the Gene Expression Omnibus (GEO) with the accession code GSE136764 [https://www.ncbi.nlm.nih.gov/geo/query/acc.cgi?acc=GSE136764]. Source data for figures 4B-D, 5E, 6C-G, and supplementary figures 8A, 8B, 8E, 9C-H, 11C and 11D are provided with the paper.
A list of publicly available datasets used in this study are listed in Supplementary Table 8. Additionally, the following public databases were used: GENCODE v26 [https://www.gencodegenes.org/human/release_26.html], FANTOM5 [https://fantom.gsc.riken.jp], Dfam [https://dfam.org/home], HOCOMOCO v11 [https://hocomoco11.autosome.ru].

Field-specific reporting

Please select the one below that is the best fit for your research. If you are not sure, read the appropriate sections before making your selection.

☒ Life sciences ☐ Behavioural & social sciences ☐ Ecological, evolutionary & environmental sciences

For a reference copy of the document with all sections, see nature.com/documents/nr-reporting-summary-flat.pdf

Life sciences study design

All studies must disclose on these points even when the disclosure is negative.

Sample size	The number of AML patient samples analysed was conditioned by the availability of public datasets.<br><br>For functional assays using CRISPR-mediated genetic and epigenetic editing experiments, sample sizes were chosen based on published results from similar experiments from our lab (PMID: 31012843) and others (PMID: 30070637, 31006620).
Data exclusions	No data were excluded.
Replication	Findings related to open chromatin at ERVs were replicated across three different cell lines and in two independent cohorts of primary samples.<br><br>Histone ChIP-seq data showed similar patterns across primary patient samples and four different cell lines, with some variability expected from the heterogeneity of AML, consistent with previous studies.<br><br>A minimum of three independent clones were analysed in CRISPR experiments, with reproducible outcomes.<br><br>CRISPRi was performed in two different cell lines, with similar phenotypic outcomes.<br><br>The effects of APOC1-LTR2 inactivation were consistent between CRISPRi, plasmid-based CRISPR and lentiviral CRISPR, and across two different cell lines.
Randomization	Randomization is not relevant, as no treatments were assigned to different subjects.
Blinding	Blinding was not used, as all data produced was from objective quantitative methods.

Reporting for specific materials, systems and methods

We require information from authors about some types of materials, experimental systems and methods used in many studies. Here, indicate whether each material, system or method listed is relevant to your study. If you are not sure if a list item applies to your research, read the appropriate section before selecting a response.

Materials & experimental systems

n/a	Involved in the study	
☐	☒	Antibodies
☐	☒	Eukaryotic cell lines
☒	☐	Palaeontology
☒	☐	Animals and other organisms
☒	☐	Human research participants
☒	☐	Clinical data

Methods

n/a	Involved in the study	
☐	☒	ChIP-seq
☐	☒	Flow cytometry
☒	☐	MRI-based neuroimaging

Antibodies

Antibodies used	Cas9 antibody (Diagenode #C15200229-100), H3K27ac antibody (Active Motif #3913), H3K9me3 antibody (Diagenode#C15410193).
Validation	Cas9 antibody (https://www.diagenode.com/en/p/crispr-cas9-monoclonal-antibody-100-ug), H3K27ac antibody (https://www.activemotif.com/catalog/details/39135), H3K9me3 antibody (https://www.diagenode.com/en/p/h3k9me3-polyclonal-antibody-premium-50-mg)

Eukaryotic cell lines

Policy information about cell lines

Cell line source(s)	OCI-AML3, MOLM-13 and HL-60 cell lines were provided by Professor Brian Huntly, originally sourced from DSMZ.<br>K562 cell line was provided by Dr Farideh Miraki-Moud, originally derived by Lozzio & Lozzio (PMID: 163658).<br>HEK293T cell line was provided by Dr. Ana O'Loghlen, originally derived in the lab of Michele Calos (PMID: 3031469).
Authentication	Cell lines were not authenticated.
Mycoplasma contamination	K562 and OCI-AML3 cell lines were tested negative for mycoplasma. HL-60 and MOLM-13 were not tested.
Commonly misidentified lines<br>(See ICLAC register)	No commonly misidentified cell lines were used.

ChIP-seq

Data deposition

☒ Confirm that both raw and final processed data have been deposited in a public database such as GEO.

☒ Confirm that you have deposited or provided access to graph files (e.g. BED files) for the called peaks.

Data access links<br>*May remain private before publication.*	https://www.ncbi.nlm.nih.gov/geo/query/acc.cgi?acc=GSE136764
Files in database submission	Raw fastq files, Broadpeak files and Bigwig track files.
Genome browser session<br>(e.g. UCSC)	https://genome.ucsc.edu/s/mbranco/AML_ERVs

Methodology

Replicates: Duplicate ChIP-seq experiments were performed for H3K27ac (K562 and OCI-AML3) and H3K9me3 (K562) LTR2B CRISPRi experiments. For all remaining samples, one replicate was sequenced.

Sequencing depth:

Sample:	Read count:
HL-60_H3K27ac	42164835
HL-60_Input	27097144
Molm13_H3K27ac	34975107
Molm13_Input	18955819
OCI-AML3-empty_H3K27ac	45904772
OCI-AML3_LTR2BgRNAs_H3K27ac-1	44716424
OCI-AML3_LTR2BgRNAs_H3K27ac-2	53152894
OCI-AML3_empty_H3K9me3	54434074
OCI-AML3_LTR2BgRNAs_H3K9me3-1	56001564
OCI-AML3_LTR2BgRNAs_H3K9me3-2	25844346
OCI-AML3_Input	15625705
K562-empty_H3K27ac	47696761
K562_LTR2BgRNAs_H3K27ac-1	52733589
K562_LTR2BgRNAs_H3K27ac-2	44289180
K562_empty_H3K9me3	27083662
K562_LTR2BgRNAs_H3K9me3	36798980
K562_empty_dCas9	30867854
K562_LTR2BgRNAs_dCas9	166807135
K562_Input	50120809

Antibodies	Cas9 antibody (Diagenode #C15200229-100), H3K27ac antibody (Active Motif #3913), H3K9me3 antibody (Diagenode#C15410193).
Peak calling parameters	Adaptors and low-quality base calls were trimmed using trim_galore! v0.4.5 with default parameters.<br>Trimmed reads were mapped to hg38 using bowtie2 v2.3.4 with default paramaters. Uniquely aligned reads were extracted using a custom perl script.<br>Peaks were called using MACS2 v2.1.1 with -q 0.05; --broad was used for histone modifications
Data quality	FastQC was used to assess raw data quality. Peak detection was performed with a q-value cut-off of 0.05.
Software	MACS2 v2.1.1 for peak detection. Other custom scripts are available at https://github.com/MBrancoLab/Deniz_2019_AML.

Flow Cytometry

Plots

Confirm that:

- [x] The axis labels state the marker and fluorochrome used (e.g. CD4-FITC).
- [x] The axis scales are clearly visible. Include numbers along axes only for bottom left plot of group (a 'group' is an analysis of identical markers).
- [ ] All plots are contour plots with outliers or pseudocolor plots.
- [x] A numerical value for number of cells or percentage (with statistics) is provided.

Methodology

Sample preparation	Cell cycle assay was performed using muse cell cycle kit by following manufacturers instructions (Millipore). For apoptosis assay, the cells were stained by an annexin V 647 (Thermofisher Scientific) and DAPI.
Instrument	BD FACS Canto II
Software	BD FACSDiva Software v6.1.3
Cell population abundance	Cell population abundance of Annexin V stained cells is indicated in Figure 6G. The bar plot indicates the average percentage of Annexin V positive cell population in control cells with no gRNA and cells with LTR2 gRNA
Gating strategy	Cells were gated on a FSC-A vs SSC-A dot-plot with debris excluded. Single cells were gated on a FSC-A vs SSC-W parameter dot-plot, with further gating on a DAPI-A vs Annexin V-AF647 dot-plot. Quadrant gating on the DAPI vs Annexin V-Af647 dot-plot allows the determination of percentage live cells (DAPI-ve/Annexin V-ve), early apoptosis (DAPI-ve/Annexin V+ve), late apoptosis (DAPI+ve/Annexin V+ve) and necrotic (DAPI+ve/Annexin V-ve).

- [x] Tick this box to confirm that a figure exemplifying the gating strategy is provided in the Supplementary Information.
